# Tsetse fly saliva: Could it be useful in fly infection when feeding in chronically aparasitemic mammalian hosts

**Published:** 2012-09-30

**Authors:** E.O. Awuoche

**Affiliations:** 1 *School of Health Sciences, Masinde Muliro University of Science and Technology. P. O Box 190 – 50100, Kakamega. Kenya*; 2 *Kisii University College. Faculty of Agriculture and Natural Resource Management, P. O Box 408 – 40200, Kisii. Kenya*

**Keywords:** Chemotaxis, Other vector saliva proteins, Trypanosome, Tsetse salivary proteins

## Abstract

Sleeping sickness and nagana are two important diseases cuased by African trypanosomes in humans and animals respectively, in tropical african countries. A number of trypanosome species are implicated in these diseases, but it is the *Trypanosoma brucei* group that is responsible for the chronic form of sleeping sickness. During the course of this chronic infection the parasite shows a clear tropism for organs and tissues and only sporadically appears in the blood stream. Notwithstanding this feature, tsetse flies normally get infected from chronically infected apparasitemic hosts. For some pathogens like the microfilaria, it has already shown that the saliva of the vector, black fly saliva contribute to orient the pathogen to the site of the vector bite. Chemotaxis of tsetse saliva may perhaps stimulate movement of *Trypanosoma brucei* parasites from tissues to the bloodstream and via the vascular to the tsetse feeding site, and could explain the relatively high infection rate of tsetse flies feeding on chronically infected animals. This review paper looks into the possible role of trypanosome-vector saliva in ensuring parasite acquisition and its application in the tsetse – trypanosome interaction at the host skin interphase.

## Introduction

African trypanosomiasis is a complex fatal disease of both human and livestock in many rural areas of sub-Saharan Africa. The disease is caused by a blood-borne unicellular flagellated protozoan parasite of the genus *Trypanosoma*, dwelling in various body tissues and fluids. Trypanosomes are motile due to the undulatory motion of their flagellum (Baral, 2010).

In human, the parasite causes sleeping sickness (also called Human African Trypanosomiasis or HAT) which can either be acute or chronic depending on the trypanosome species involved. The acute form of HAT is caused by *T. brucei rhodesiense* in eastern and southern Africa while the chronic form is caused by *T. brucei gambiense* in western and central Africa (Giroud *et al.*, 2009). Currently however, there is an insurgence and an increasing potential of overlap between chronic and acute infections in East Africa, especially in Uganda (Welburn *et al.*, 2001; Picozzi *et al.*, 2005; Batchelor *et al.*, 2009; Wardrop *et al.*, 2010).

In animals, the disease is called “nagana” (Animal African Trypanosomiasis or AAT) and is caused by a number of trypanosome species. Both the parasites of HAT and AAT are carried and cyclically transmitted by the tsetse fly, *Glossina spp*, which are obligate blood feeding insects (Aksoy *et al.*, 2003). Other trypanosome species also exist and cause infections outside Africa like *T. evansi*, the causative agent of ‘surra’ in central and southern America, the Middle East and Asia (Baral, 2010). African trypanosomiasis has no vaccine and leads to mortality if left untreated. In addition, the current regime of drugs used in the second phase of HAT infection are highly toxic and there is now evidence of the rapid development of drug resistance in trypanosomes during the treatment of AAT (Delespaux *et al.*, 2008; Claes *et al.*, 2009; Giroud *et al.*, 2009).

Moreover, the present diagnostic tools are based on parasite detection and the detection of antibodies produced against the conserved variable surface glycoprotein (VSG) of trypanosome. These diagnostic tools are however, limited in sensitivity for low parasitemic, chronic and silent infections (Paris *et al.*, 1982; Giroud *et al.*, 2009).

The disease has a complicated epidemiology especially with the involvement of wildlife and domestic animals (cattle, sheep, goats and pigs) as reservoir of the zoonotic HAT parasite (*T. b. rhodesiense*). This affects transmission and spread of the disease bringing the parasite closer to the human residence (Fevre *et al.*, 2001; Welburn *et al.*, 2001; Ng’ayo *et al.*, 2005).

This may be due to the fact that domestic animals present no clinical signs when infected by these trypanosomes, despite having parasitic load enough to infect the vector. The epidemiology and transmission of HAT is equally affected by a complex interrelationship between man, parasite and tsetse fly as well as a number of environmental factors (Kuzoe, 1989).

In man and other vertebrates, the skin is the most important site for the host-parasite-vector interplay and for pathogens; it forms an ecologically privileged site to exploit (Nuttall and Labuda, 2004). It is an area that pathogens use to gain entry into either the host or the vector upon modification by the vector’s saliva. Tsetse flies, like other arthropods can transmit and acquire parasites during feeding time. Their saliva plays a crucial role in the feeding process and parasite transmission and in response, host hemostasis and immunity is triggered and inflammatory cells are recruited to the bite site to disrupt feeding (Ribeiro and Francischetti, 2003; Caljon *et al.*, 2006b).

To overcome this host reaction, tsetse fly injects its pharmacologically active anticoagulants and immunosuppressive salivary proteins (Cappello *et al.*, 1996; Ribeiro and Francischetti, 2003; Caljon *et al.*, 2006b, 2010) which also biases the host immune responses to T – helper type 2 (Th2) (Caljon *et al.*, 2006a).

Previous studies on vector-borne diseases focused on pathogen movement from the vector to the vertebrate host. In these studies, there is evidence that arthropod saliva promotes and rapidly increases parasite transmission and infection to the host (Jones *et al.*, 1989; Caljon *et al.*, 2006b), multiplication and distribution in the host (Horka *et al.*, 2009) and transmission between infected and non-infected vectors feeding on a non-infected animal without the pathogen getting circulated in the peripheral vascular system (Gern and Rais, 1996; Patrican, 1997). In addition, the survival of pathogens during their transmission also depends on their ability to exploit the pharmacological activities of salivary protein molecules (Nuttall and Labuda, 2004; Horka *et al.*, 2009).

However, even though very little is known about the opposite process, it is postulated that pathogens may also require vector-salivary proteins to be transmitted into their biological vectors (Nuttall and Labuda, 2004). This has, been demonstrated in few research studies: for example, in ticks, it has been demonstrated that in *Ixodes scapularis*. Salivary gland genes *Salp1*6 and *Salp25D* are required for acquisition of *Anaplasma phagocytophilum* and *Borrelia burgdoferi* parasites respectively (Sukumaran *et al.*, 2006; Narasimhan *et al.*, 2007).

The salivary Microfilarial Orientation Factors (MOF) of black fly saliva is also known to direct the movement of microfilariae larvae in the mammalian skin to the site of the fly bite. Consequently, high rate of black fly infection has been reported in the field even when they feed on people with low levels of microfilariae in their skin (Stallings *et al.*, 2002).

In tsetse flies, the transmissibility of *T. brucei* and *T. congolense* parasites from the host to the vector has been shown to be independent of the level of parasitemia in the animals (Moloo *et al.*, 1999; Van den Bossche *et al.*, 2005; Akoda *et al.*, 2008).

Despite the fact that parasites of *T. brucei* species invade and inhabit other organs and extra-vascular tissue spaces other than blood vessels (Losos and Ikede, 1972; Claes *et al.*, 2009; Giroud *et al.*, 2009) they can still easily infect the fly (Moloo *et al.*, 1999; Van den Bossche *et al.*, 2005). However, for *T. vivax*, acquisition by tsetse fly tends to depend on the level of parasitemia (Moloo *et al.*, 1999).

Initial studies showed that tsetse flies readily get infected when they feed on a blood meal containing 50-20,000 trypanosomes per mm^3^ and 10-20,000 short stumpy forms per mm^3^ (Page, 1972). However, these findings were contradicted in recent work with *T. brucei* infection in cattle in Uganda and Kenya showing that there is no substantial difference in the fly infection rates when fed on acute and apparently chronic aparasitemic infected cattle (Moloo *et al.*, 1999; Van den Bossche *et al.*, 2005).

The ease with which tsetse flies can get infected when feeding on these apparent aparasitemic infected animals poses a great threat to human health. This suggests that there may be a mechanism causing an increased level of parasitaemia at the tsetse feeding site, possibly some kind of chemo-attractant in the tsetse saliva injected intra-dermally during feeding. This phenomenon, and whether trypanosomes move to the point of tsetse bite in the skin as a response to an environmental cue or chemotactic signals from the fly salivary protein deposited during fly feeding, remains a matter of conjecture.

This review looks into the probability of chemo-attraction of tsetse salivary protein and will borrow much from other vector-borne diseases. This is important in understanding the epidemiology and biology HAT and AAT parasites to enable the development and implementation of more suitable and effective control measures.

### Life cycle and biology of trypanosomes

Trypanosome parasites alternate their life cycle between vertebrate and invertebrate hosts. Transmission is assured by tsetse flies and to a lesser extent by other blood sucking flies (Hoare, 1972). The parasites differentiate in distinct life stages in order to prepare for and adapt to different environments they encounter during their life cycle (MacGregor and Matthews, 2010). Upon successful establishment of the *T. brucei* parasites in a vertebrate host, the blood stream form *T. brucei* form trypomastigotes show clear tropism to body other tissues and organs (Claes *et al.*, 2009; Giroud *et al.*, 2009) which may lead to very low undetectable parasitemia in chronic infections.

Despite this feature, tsetse flies still manage to infect themselves when feeding on these aparasitemic hosts (Moloo *et al.*, 1999; Van den Bossche *et al.*, 2005). With this observation, it is reasonable to argue that as tsetse fly probe for a blood meal, it deposits a pool of saliva at the point of feeding which may contain chemo-attractant proteins to trypanosomes enabling directed mobility to feeding site.

It is also possible to reason that tsetse may inject its saliva directly into the vascular system using its long proboscis, then as saliva get transported in the vascular system their chemo-attractant proteins could attract parasite into the bloodstream enabling uptake. When trypanosomes are taken up by the vector, they complete their complex two to three weeks life cycle in the fly (Chappuis *et al.*, 2005; Baral, 2010).

The development in tsetse fly differs between trypanosome species: *Trypanosoma vivax* is restricted to the mouthparts and has a short life cycle in the fly compared to *T. congolense* and *T. brucei* that develop in the mid-gut and mature in the mouthparts or in the salivary glands, respectively (Woolhouse *et al.*, 1994; Aksoy *et al.*, 2003). Colonization of the salivary gland by *T. brucei* species takes place within a limited time period in the salivary gland region (Van Den Abbeele *et al.*, 1999). It is however not known what happens to parasites which have not moved to the salivary gland. Once infected, the flies transmit trypanosomes for life (Mshelbwala, 1972; Aksoy *et al.*, 2003).

### Distribution and pathogenesis of African trypanosomes within the vertebrate host

In the vertebrate host, different trypanosome species, strains and clones invade different tissues and organs, enabling division into haematic and humoral groups. The haematic group seems confined to the vascular system while the humoral group has capacity to penetrate the capillary endothelium and multiply in the extra-vascular tissue spaces (Losos and Ikede, 1970, 1972; Goodwin, 1971; Ojok *et al.*, 2002).

The trypanosomes of the *T. brucei* species group belong to the humoral group and get widely distributed in connective tissue fluids, lymph, cerebrospinal fluid, and even aqueous humor of the host (Losos and Ikede, 1972; Tizard *et al.*, 1978). Current bioluminescence imaging studies using infected mice with *T. brucei* specie have shown that these parasites invade other organs like the brain, testis, lungs, liver, and spleen (Claes *et al.*, 2009; Giroud *et al.*, 2009).

The haematic groups of *T. congolense* nor *T. vivax* species however do not leave the bloodstream (Losos and Ikede, 1970, 1972; Ojok *et al.*, 2002). They stay in microcirculation attached to the capillary vascular endothelium (Losos and Ikede, 1972; Banks, 1978; Ojok *et al.*, 2002) leading to 5 to 10 times greater concentration in capillary beds than in large vessels (Tizard *et al.*, 1978); a feature that may enable them be easily taken up by fly during bloodmeal.

Variation in the distribution of trypanosomes species within the mammalian host lead to differences in their pathogenesis (Losos and Ikede, 1970). These two groups based on their features are expected to show variation in transmissibility. However, studies have shown no difference in transmissibility (Moloo *et al.*, 1999; Van den Bossche *et al.*, 2005; Akoda *et al.*, 2008), and such likelihood still remains a question.

### Motility of African trypanosome parasites

To invade extravascular tissues and organs, African trypanosomiasis parasites should be able to exhibit active motility. Like their relatives in the order *Kinetoplastida*, they use their undulating flagella for motility and can move at an approximate speed of 20μms^-1^ through traction by their flagella in between the cells in liquid medium (Oberholzer *et al.*, 2007; Ginger *et al.*, 2008; Ralston *et al.*, 2009).

The previous spiral motility of trypanosomes, reminiscent of chemotaxis (Hill, 2003; Branche *et al.*, 2006; Baron *et al.*, 2007) has currently been improved by Uppaluri *et al*. (2011) as they showed that straighter trypanosomes swim more directionally compared to tumblers. Being extracellular parasites, trypanosomes highly depend on their own motility power to migrate in both their hosts. In tsetse fly, importance of active parasite motility still needs to be investigated.

This ability to move is believed to be a major factor in parasite pathogenicity in mammalian hosts and parasite development in tsetse flies where they undergo directed migration to the salivary gland for the completion of their developmental stages to infective mature metacyclic trypomastigotes (Roditi and Lehane, 2008; Ralston *et al.*, 2009).

In tsetse fly, the blood stream parasites changes to procyclic trypanosomes. Here, they undergo complex series of developmental transformations and directional migrations to complete their development into mammalian infectious forms in the tsetse salivary glands. Recent studies have shown that specific fly tissues get heavily infected, whereas adjacent tissues are devoid of parasites, indicating that parasite migration is not random (Gibson and Bailey, 2003; Gibson *et al.*, 2006).

The invasion of the salivary gland by the parasites from the foregut and proboscis occurs within a limited time period during the developmental phase (Van Den Abbeele *et al.*, 1999).

Previously, it was thought that it is the asymmetrically dividing epimastigotes that move to the salivary gland where they accomplish their division to short epimastigotes (Van Den Abbeele *et al.*, 1999; Hill, 2003). However, recent study indicate that asymmetric division of epimastigotes take place prior to entry to the salivary gland; and it is the short epimastigotes that are seen attached and free in the salivary gland (Sharma *et al.*, 2008).

In principle, it could have been reasonable to find long epimastigotes in the salivary gland due to their ability to move (Van Den Abbeele *et al.*, 1999). This however is not the case and it is not clear how the short epimastigotes gain entry into the salivary gland (Sharma *et al.*, 2008) as motility has not been demonstrated in them. Whether they are the ones exhibiting tumbling motility (Uppaluri *et al.*, 2011) in response to tsetse salivary protein in the fly needs to be investigated.

In mammals, trypanosomes penetrate the vascular wall and the microvascular endothelium of the blood brain barrier (BBB) (Nikolskaia *et al.*, 2006a, 2006b; Masocha *et al.*, 2007; Ralston *et al.*, 2009). In cattle, both *T. congolense* and *T. vivax* are known intravascular parasites, while *T. b. brucei* can leave the blood vessels and invade other tissues in cattle, but not usually the central nervous system (CNS) (Naessens, 2006). However, about half of all cattle infected with *T. b. rhodesiense* develop fatal CNS disease. In addition, *T. b. gambiense* has been demonstrated to cross an *in vitro* model of the human BBB more efficiently compared to *T. b. brucei* (Grab *et al.*, 2004). Collectively, these data show that *T. b. gambiense* and *T. b. rhodesiense* are truly CNS tropic organisms.

Moreover, recent studies have also shown tropism of *T. brucei* species group to the testis crossing blood testis barrier (Claes *et al.*, 2009; Giroud *et al.*, 2009). African trypanosomes are digenetic parasites (Parsons and Ruben, 2000) and undergo directional motility. As such they must integrate both host and parasite derived signals in order to be successful in establishing within a specific host compartment (Oberholzer *et al.*, 2007, 2010; Ginger *et al.*, 2008).

In order to thrive in hosts, trypanosomes must actively search for the correct chemical environment to stay. This can be by the use of their biochemical receptors in their external membrane which can enable them to detect external signals and direct their movement towards the gradient of attractive substance (Pozzo *et al.*, 2009). Previous studies show that for African trypanosomes to cross the human BBB, it involves paracrine signaling between parasite and host (Nikolskaia *et al.*, 2006a, 2006b).

As in the flagellum of other organisms, the flagella of trypanosomes have localized cyclic nucleotide and calcium signaling pathways implicating the flagella to have a sensory role in detecting environmental cues (Hill, 2003; Ralston *et al.*, 2009). Additionally, African trypanosomes genes encode various classical signal transduction pathway proteins (Parsons and Ruben, 2000).

Moreover, several uncharacterized gene families with predicted cell surface proteins, some of which that no known functions have been determined. These may probably serve sensory and perhaps recognition roles (Fragoso *et al.*, 2009; Jackson *et al.*, 2010). Also, some evolutionary conserved genes of *T. brucei* have been identified to form components of flagella which are necessary for parasite motility (Broadhead *et al.*, 2006; Baron *et al.*, 2007).

Recent *in vitro* studies have shown that trypanosomes engage in flagellum-mediated social motility where by the parasites assemble into multicellular communities with emergent properties that are not evident in single cells (Oberholzer *et al.*, 2010).

Parasites in these groups are shown to undergo polarized migrations and cooperate to divert their movements in response to external signals. Altogether, their ability to sense external signals and react to them and capability to move could be the mechanism by which trypanosomes maneuver their way to the point of saliva deposit during tsetse feeding in response to tsetse salivary protein.

### The tsetse fly saliva and its proteins

Tsetse saliva is a relatively alkaline (pH=8.0) fluid with over 20 (and over 250 for *G. m. morsitans*) salivary proteins that are probably involved in blood feeding process. The saliva forms the medium in which trypanosomes thrive as they undergo metacyclic developmental stages in the fly and also act as a “fluid vehicle” through which mature (metacyclic) infective trypanosomes are transported into the vertebrate host (Li *et al.*, 2001; Van Den Abbeele *et al.*, 2007; Alves-Silva *et al.*, 2010).

Generally, each salivary gland of tsetse flies e.g. *G. m. morsitans* harbours about 4.3 - 5.0 μg of soluble proteins of which approximately 50% (about 4.0μg from the paired glands) is injected with the saliva during blood meal. After the blood meal, the total protein content of the fly’s salivary gland is at its lowest but increases significantly within 48hours. However, some major salivary genes (*TAg5* and *Tsal1&2*) are continuously expressed but are only moderately up-regulated two days after blood feeding (Caljon *et al.*, 2006a; Van Den Abbeele *et al.*, 2007).

In the naive tsetse salivary gland micro-environment, there are saliva components that enhance the infection onset upon trypanosome inoculation in the host skin (Caljon *et al.*, 2006b).

Other saliva constituents are essential for the hematophagous behavior of the tsetse fly by counteracting host responses such as vasoconstriction, platelet aggregation and coagulation reactions involving serine proteases such as thrombin (Ribeiro and Francischetti, 2003). A number of saliva proteins are implicated in facilitating blood feeding: tsetse thrombin inhibitor (TTI) (Cappello *et al.*, 1996, 1998) and salivary apyrases such as 5’nucleotidase related protein, Glossina morsitans morsitans salivary gland protein 3 (Sgp3) including at least one with fibrinogen receptor (GPIIb/IIIa) antagonistic properties (5’Nuc) (Caljon *et al.*, 2010); these may also enable vector infection when feeding on infected host.

Other abundant salivary components include putative endonucleases like tsetse salivary gland proteins 1 and 2 (Tsal1 and Tsal2) (Li *et al.*, 2001), putative adenosine deaminases; tsetse salivary gland growth factors 1 and 2 (TSGF-1 and TSGF-2) (Li and Aksoy, 2000) and an antigen5-related allergen; tsetse Antigen5 (TAg5) (Caljon *et al.*, 2009).

An insight into the *G. m. morsitans* sialome by Alves-Silva *et al*. (2010) identified many other tsetse salivary proteins. Most of these saliva proteins however have no known functions (Li *et al.*, 2001; Van Den Abbeele *et al.*, 2007; Alves-Silva *et al.*, 2010). There could be a possibility that one or many of these identified tsetse saliva proteins (with unknown functions) may have chemo-attraction properties sensed by trypanosomes.

### Role of vector saliva in transmission, proliferation and distribution of parasites in vertebrate host

Vector-borne parasites transmission to vertebrate hosts is influenced by arthropod vector saliva injected with parasites in the host tissue. In addition to saliva protein’s immunomodulatory properties, their vasodilatory effects influence the dynamics of blood feeding in such a way that they may affect parasite transmission (Champagne, 1994).

In tsetse fly, saliva has been shown to induce Th2-biased immune response and reduces the host’s inflammatory response (Caljon *et al.*, 2006a). Consequently, tsetse fly saliva and the functional Antigen-5 related allergen (linked to local hypersensitivity reaction) in saliva may lead to rapid and early onset of trypanosome infection (Caljon *et al.*, 2006a, 2006b, 2009). Similarly, studies conducted using sand fly saliva showed modulation of the host immune responses supporting the initiation of *Leishmania* infection (Titus and Ribeiro, 1988; Hall and Titus, 1995; Lima and Titus, 1996; Belkaid *et al.*, 1998).

Monocytes have been demonstrated to be attracted by the salivary gland extract of *Phlebotomus duboscqi*, the vector for *Leishmania* parasites. This can consequently lead to early infection since *Leishmania* parasites are intracellular pathogens that infect macrophages and may take this advantage for successful host parasitisation. In addition, the inhibitory effect of sand fly saliva on macrophage antigen presentation and interferon gamma (IFN-γ) activation enables successful infection establishment (Anjili *et al.*, 1995). Saliva of other arthropods, such as ticks, black flies and mosquitoes, has similar effects (Mejri *et al.*, 2001; Stallings *et al.*, 2002; Schneider *et al.*, 2004). Parasites also exploit various salivary proteins for their effective transmission (Foley and Nieto, 2007).

To demonstrate the effect of saliva proteins on parasite transmission, Ramamoorthi *et al*. (2005) used Salp15 protein of *Ixodes ricinus* tick and showed that it increased the transmission of *B. burgdorferi* spirochetes in mammalian host. Moreover, saliva also increases the multiplication and distribution of parasites in the mammalian host. This has been demonstrated for *Ixodes ricinus* tick (Horka *et al.*, 2009).

### Role of vector saliva in parasites uptake by the vector

Successful acquisition of parasites by vectors from the infected vertebrate host may be also assured by the saliva proteins they inject (Nuttall and Labuda, 2004). The tick salivary proteins, Salp25D (Anderson and Valenzuela, 2007; Narasimhan *et al.*, 2007) and Salp16 (Sukumaran *et al.*, 2006) of *Ixodes scapularis* have been demonstrated to play critical roles in *B. burgdorferi* and *A. phagocytophilum* vector acquisition respectively. RNA interference – mediated silencing of *Salp25D* and *Salp16* genes expression interfered with acquisition of the two parasites respectively (Sukumaran *et al.*, 2006; Anderson and Valenzuela, 2007; Narasimhan *et al.*, 2007).

In black fly, *Onchocerca lienalis* microfilariae has been shown to be attracted by the MOF of fly salivary gland (Stallings *et al.*, 2002). Therefore, it is thought that MOF of black fly saliva serve as a means used by microfilariae to find and infect the fly during blood-feeding and as it induces movement and orientation of microfilariae to the site of biting. Strong (1931) noted the large numbers of microfilariae found at the vector feeding site on the skin soon after the blood meal which could have been due to the black fly salivary MOF.

The chemo-attraction of vertebrate monocytes to the salivary gland extract of sand flies (Anjili *et al.*, 1995) can also be a means through which the vector gets infected with *Leishmania* parasites. The vasodilatory effect of arthropod saliva that increases the flow of blood to the feeding site may also lead to more parasites moving to that point and leading to parasites acquisition by the feeding vectors. Saliva has also been demonstrated to facilitate the transmission of viruses and pathogens between infected and non-infected vectors feeding together (co-feeding) via non-systemic route (Gern and Rais, 1996; Patrican, 1997; Mead *et al.*, 2000).

In African trypanosomes, studies have shown that animals chronically infected with *T. brucei* species may present a fluctuating parasitaemia that may be difficult to diagnose with the routinely used parasitological tests (Van den Bossche *et al.*, 2005). This could be due to parasites invading other organs and tissues that are immuno – privileged to escape the host immunity.

These animals however, when fed on by tsetse flies, infection rates in the flies are surprisingly high (Moloo *et al.*, 1999; Van den Bossche *et al.*, 2005). For the procyclic trypanosomes, *in vitro* studies indicate that they form colonies on the surface of agarose which are able to move *en masse* as a large single group across the agarose surface in response to an external stimulus (Oberholzer *et al.*, 2010). *In vitro* chemotaxis of *Trypanosoma musculi* to macrophages in agarose (Samarawickrema and Howell, 1988), as well as for other parasites to cells (Nelson *et al.*, 1975; Chenoweth *et al.*, 1979; Fischer and Czarnetzki, 1982) has been demonstrated.

Based on the trypanosome directed movement in the fly to the salivary gland and heavy colonization of specific parts of the salivary gland epithelium, trypanosomes may respond to chemo-attractants in the salivary gland and undergo chemotaxis (Van Den Abbeele *et al.*, 1999; Oberholzer *et al.*, 2007; Uppaluri *et al.*, 2011).

In mammals, the presence of diffusible salivary chemo-attractants that elicits an immediate movement orientation response of trypanosomes is especially important to *T. brucei* species extravascular parasites. This is important in ensuring “economy of movement” of the parasites (Losos and Ikede, 1972; Masocha *et al.*, 2007). The spread of these chemo-attractants in the saliva from the bite point through the extra-vascular matrix of the skin can also be supported by the salivary hyaluronidase enzyme that has currently been partially described (Alves-Silva *et al.*, 2010).

Due to the fact that trypanosomes are digenetic parasites, they must be able to integrate both host and parasite signals in order to complete their life cycle (Parsons and Ruben, 2000; Oberholzer *et al.*, 2010). The genome of trypanosome encodes several signal transduction pathway proteins as well as numerous proteins on their surface many of which have no known functions. There are predictions that these surface proteins of unknown functions may have sensory or signaling roles (Parsons and Ruben, 2000; Fragoso *et al.*, 2009; Oberholzer *et al.*, 2010).

These surface signaling proteins, together with the undulating flagella (a highly complex organ with numerous proteins), may aid in detection of chemical cues in the saliva and the orientation of trypanosome movement (Hill, 2003; Baron *et al.*, 2007; Oberholzer *et al.*, 2007; Ginger *et al.*, 2008; Ralston *et al.*, 2009).

In principle, if these sensory signaling surface proteins enable detection of chemical cue and elicit directed movement of trypanosomes, then this chemical interplay between specific vector-secreted saliva components and the sensory receptors of the parasite take place in a biochemical milieu within the mammalian host skin.

Contrary to the effect of saliva on parasite acquisition, there are vector and parasite intrinsic factors that affect transmission of trypanosomes. The tsetse fly takes a blood meal about two times its unfed body weight (Lehane, 2005) which may increase the chances of the fly picking parasites during feeding. Additionally, tsetse flies have fairly long mouth parts that can easily enable them take a blood meal directly from the blood vessels.

The parasite transmissibility have also been shown to depend on the virulence of parasite (Masumu *et al.*, 2006), resistance of parasite to trypanocidal drugs (Van den Bossche *et al.*, 2006) and on various isolates (Ravel *et al.*, 2006). It is therefore important to eliminate such factors before reaching the conclusion that tsetse saliva may contain some chemo-attractant for the trypanosome.

### Perspectives and comments

Substantial progress has been made in recent years in understanding trypanosome transmission at the level of basic biology. However, these studies have been directed to understanding the movement of parasites from the vector to the vertebrate hosts with only very few in the reverse direction. The modern development in biomedical science and molecular biology technologies and *in vitro* techniques could be important illustrating the role of tsetse saliva in the transmissibility of trypanosomes.

The *in vitro* technique using semi-solid agarose could be the most appropriate method to illustrate real chemotaxis (movement) of trypanosomes in response to tsetse saliva. A number of *in vitro* techniques have been used to study chemotaxis of parasites of *Leishmaniasis* (Ahmed *et al.*, 1998; van *et al.*, 2002; Barros *et al.*, 2006; Pozzo *et al.*, 2009;), *Plasmodium sporozoites* (Akaki and Dvorak, 2005) and *Onchocerca lienalis* microfilariae (Lehmann *et al.*, 1995; Stallings *et al.*, 2002). These systems employ the use of a membrane and offer several ways to demonstrate chemotaxis. Importantly, the use of genetically modified *Renilla* Luciferase expressing trypanosome with the capability to produce light in the bioluminescent machine could provide a simple way to reveal real time mobilization of trypanosome in response to tsetse saliva. If tsetse saliva is useful in mobilization of trypanosomes, it offers great potential for improving knowledge in new ways of considering the epidemiology of African trypanosomiasis and its control.

As it currently stands, prevention and control of both HAT and AAT are based on the vector control and treatment of infected humans and animals using their respective trypanocidal drugs (Baral, 2010). Due to this increasing development of trypanocidal resistance and absence of efficient vaccines, alternative control measures should be explored.
